# Evaluation of the efficient propagation of *Rhizophagus intraradices* and its inoculation effects on rice

**DOI:** 10.1128/aem.00558-25

**Published:** 2025-06-24

**Authors:** Feng Shi, Xinghao Wang, Xue He, Tianle Xu, Mingguo Jiang, Wei Chang, Fuqiang Song

**Affiliations:** 1 Engineering Research Center of Agricultural Microbiology Technology, Ministry of Education & Heilongjiang Provincial Key Laboratory of Ecological Restoration and Resource Utilization for Cold Region & Key Laboratory of Microbiology, College of Heilongjiang Province & School of Life Sciences, Heilongjiang University 12432, Harbin, China; 2 School of Marine Sciences and Biotechnology, Guangxi Key Laboratory of Polysaccharide Materials and Modifications, Guangxi Minzu University 47874 , Nanning, China; Norwegian University of Life Sciences, Ås, Norway

**Keywords:** arbuscular mycorrhizal fungi, inoculation effects, root morphology, chlorophyll fluorescence parameters

## Abstract

Arbuscular mycorrhizal fungi (AMF) are a key group of fungi closely associated with agricultural production within soil microbial communities. However, large-scale propagation of AMF inoculum faces various challenges, limiting our ability to obtain and utilize these inocula on a broad scale. To address this, we designed a monolayer mesh cultivation system employing a hydroponic approach for propagating arbuscular mycorrhizal fungi, specifically *Rhizophagus intraradices*. We conducted a comparative analysis of quality and inoculation efficiency between the water culture inoculum (w-Ri) and traditional soil-based inoculum (s-Ri). Our findings revealed the following. (i) The propagation cycle of w-Ri inoculum is 35 days and only 23% of the 150-day cycle required for s-Ri inoculum. (ii) The spore density, viability, and purity of w-Ri inoculum are 5.25 times, 1.09 times, and 1.26 times higher, respectively, than those of s-Ri inoculum. (iii) The w-Ri inoculants demonstrate effects on enhancing rice biomass, root morphology, and photosynthesis that are consistent with those of the s-Ri inoculants, while requiring only 10% of the application rate of the s-Ri inoculants. These results provide crucial theoretical references for establishing a pure and efficient arbuscular mycorrhizal fungus propagation system and its promotion and application.

**IMPORTANCE:**

The development of a monolayer mesh hydroponic cultivation system for propagating *Rhizophagus intraradices* offers a significant advancement in overcoming the challenges of large-scale AMF inoculum production, which is critical for enhancing agricultural sustainability. The comparative analysis of water culture-based (w-Ri) and traditional soil-based (s-Ri) inoculum demonstrates the superior efficiency of the w-Ri system in terms of propagation speed, spore density, and inoculum quality, highlighting its potential for large-scale application in farming practices. The findings that w-Ri inoculants are equally effective in promoting plant growth while requiring only a fraction of the application rate of s-Ri inoculants underscore the potential for reducing both cost and environmental impact in agricultural inoculation practices.

## INTRODUCTION

As the global population continues to rise annually, the demand for food production and security intensifies to meet human survival needs. Currently, the application of chemical fertilizers remains the primary strategy for enhancing grain yield (1). However, excessive reliance on chemical fertilizers can lead to the degradation of soil quality, a decline in crop quality, and various environmental risks, including non-point source pollution (2). Furthermore, due to diminishing marginal returns, the imbalance between the input and output ratios of chemical fertilizers has made it increasingly difficult for grain production to keep pace with the rate of population growth (3). Therefore, there is an urgent need to explore a green agricultural approach that can protect the environment, reduce the use of chemical fertilizers, and simultaneously enhance grain yields.

Arbuscular mycorrhizal fungi (AMF) are a type of endophytic fungus widely found in nature, capable of forming symbiotic relationships with 80% of terrestrial plants. They are among the most agriculturally relevant fungi within the soil microbial community (4). In recent years, the application of AMF in plant growth and agricultural production has garnered increasing attention. Researchers have primarily focused on exploring the interactions of mycorrhizal fungi, cultivation techniques, application effects, and their performance across different cultivation systems (5, 6). AMF not only effectively promote plant growth but also enhance plants’ resistance to environmental stress and improve nutrient absorption efficiency (7, 8). The application of AMF can also reduce the use of chemical fertilizers, enhance crop yield and quality, and improve soil structure, thereby contributing to sustainable agricultural development (9). Studies indicate that the use of homemade AMF inoculants yields significant agricultural benefits compared to commercial products (10). The use of microbial fertilizers as a replacement for traditional mineral fertilizers in soilles cultivation can also significantly enhance plant quality (11). Additionally, researchers have progressively developed models and methodologies to better assess and optimize the application effects of AMF (12, 13). However, due to the biological characteristics of AMF, their reproduction currently relies solely on the root systems of higher plants (14). Moreover, the substantial requirement for growth substrates (such as soil) during the application process limits the use of AMF in laboratory and industrial-scale settings (15). Therefore, exploring and developing propagation methods that can yield large quantities of pure AMF inoculum is of significant importance for the utilization of AMF resources and the advancement of sustainable agriculture.

Currently, there are three methods for propagating AMF. The first method is *in vitro* cultivation, which involves studying the symbiotic relationship between AMF and carrot root hairs using culture media and bioreactors. This approach has led to the development of a second-generation biological process for AMF cultivation (16). However, the *in vitro* cultivation method that relies on root hairs requires stringent experimental conditions, making it challenging for large-scale experiments and practical applications. The second method is solid substrate cultivation, which primarily includes pot cultivation and field cultivation techniques (17). This method enables the production of a large quantity of AMF inoculum by utilizing the root systems of host plants and various growth substrates such as soil, vermiculite, and coconut coir (18). Specific methods can also be employed to preserve the fungal strains, providing a resource for future research and applications. However, this propagation method faces several challenges, including high substrate demand, susceptibility to contamination by harmful microorganisms, and prolonged propagation cycles (19). The third method is liquid substrate cultivation, which utilizes water or other nutrient solutions as the growth medium for both plants and AMF. This approach significantly mitigates environmental issues associated with the use of solid substrates during AMF propagation and prevents contamination of AMF inoculum by harmful microorganisms (20). Recent studies have explored the symbiotic effects of AMF on plants using a low-cost hydroponic system, demonstrating that this system effectively promotes fungal growth while enhancing the growth rate and vigor of the plants. However, there is a scarcity of specific experimental data and comparative studies regarding the inoculum obtained from hydroponic systems. Additionally, due to technical challenges, this approach has not yet been widely adopted. Nevertheless, hydroponic technology, as a production method less influenced by environmental factors, holds significant potential for the production of AMF inoculum (21, 22). By optimizing operational processes through technological innovations, there is potential to significantly enhance production efficiency. Furthermore, diversifying cultivation methods could provide a stable and ample supply of AMF resources for agricultural cultivation and ecological restoration.

**Fig 1:**
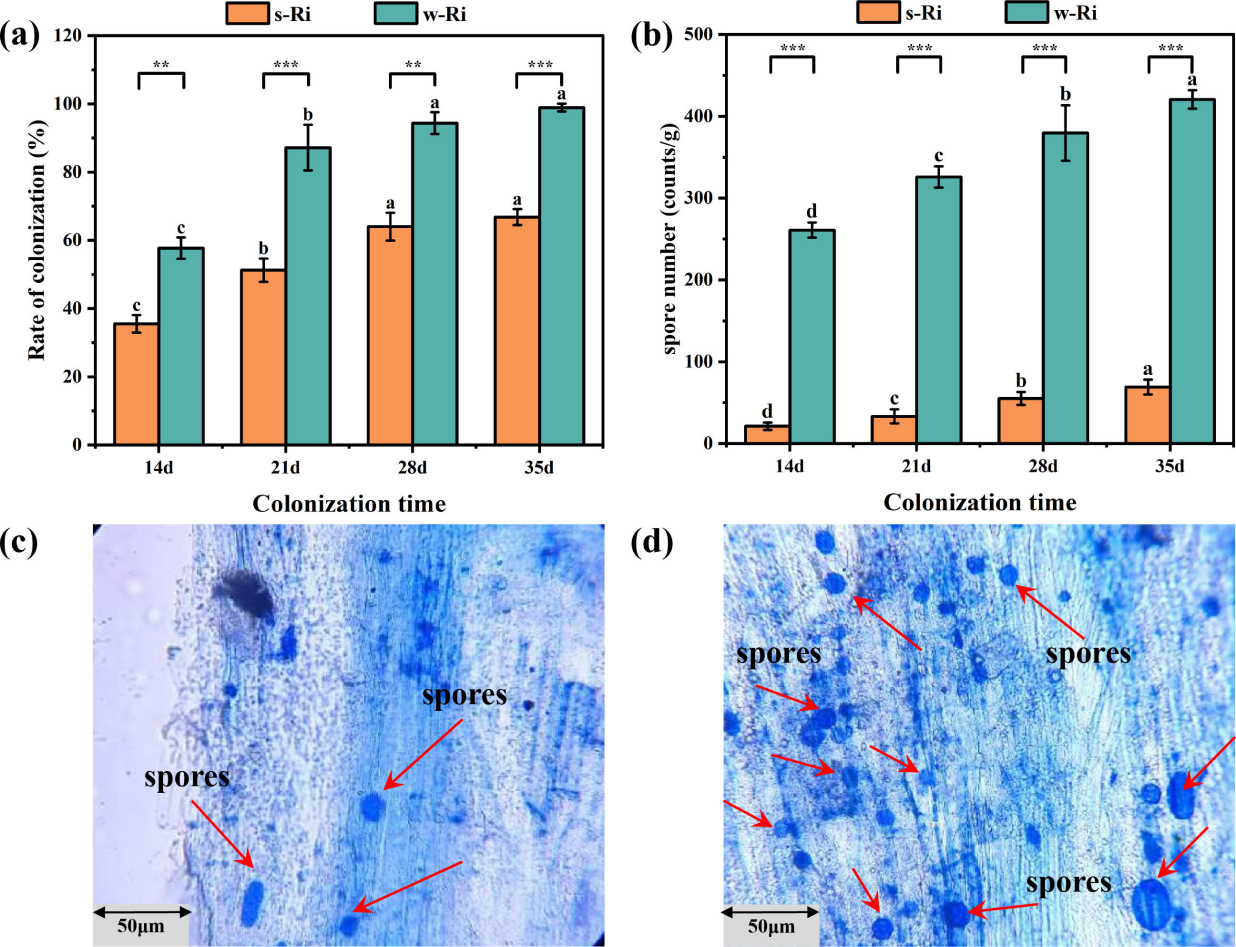
Colonization status at different time points during the propagation of Ri inoculum and the number of rhizosphere spores. (a) The colonization rates of two different Ri inoculum at various time points; (b) number of spores of two Ri inoculum at different times; (c) the spores of s-Ri inoculum; (d) the spores of w-Ri inoculum. The results are the means ± standard deviations of 5 values; there were no significant differences observed among the treatment groups containing the same letters, and * represents differences between different Ri inoculums at the same time. *P < 0.05, **P < 0.01, ***P < 0.001.

This study introduces a single-layer mesh cultivation system designed for the propagation of arbuscular mycorrhizal fungi (w-Ri) in a liquid medium. We scrutinized the quality of the inoculum derived from this system, contrasting it with the traditional soil-based inoculum (s-Ri) through quality assessment and efficacy trials of inoculation. Based on our findings, we propose the following hypotheses: (i) the w-Ri inoculum produced from the liquid culture in the single-layer mesh cultivation system surpasses the quality of the s-Ri inoculum; (ii) the incorporation of the w-Ri inoculum significantly enhances the root morphology of rice and boosts its photosynthetic capacity. These findings contribute to the data foundation for the establishment of a stable, long-term hydroponic propagation system for arbuscular mycorrhizal fungi (Ri) inoculum. Furthermore, this system potentially provides a substantial supply of AMF inoculum for agricultural production, presenting a novel method for the application of Ri inoculum Fig. 1.

## RESULTS

### Mycorrhizal colonization and sporulation of the two Ri inoculums

The mycorrhizal colonization rates of both the s-Ri inoculum and w-Ri inoculum significantly increased over time, exhibiting a consistent trend as propagation duration increased (*P* < 0.01). Notably, the mycorrhizal colonization rates of the w-Ri inoculum were significantly higher than those of the s-Ri inoculum (*P* < 0.05) (Fig. 1). In contrast, the number of spores in the w-Ri inoculum was significantly greater than that in the s-Ri inoculum (*P* < 0.001). The w-Ri inoculum contained 5.25 times more spores at day 35 compared to the s-Ri inoculum at day 150 (Fig. S1).

### Fungal propagule production and equipment capacity of the two Ri inoculums

**Fig 2:**
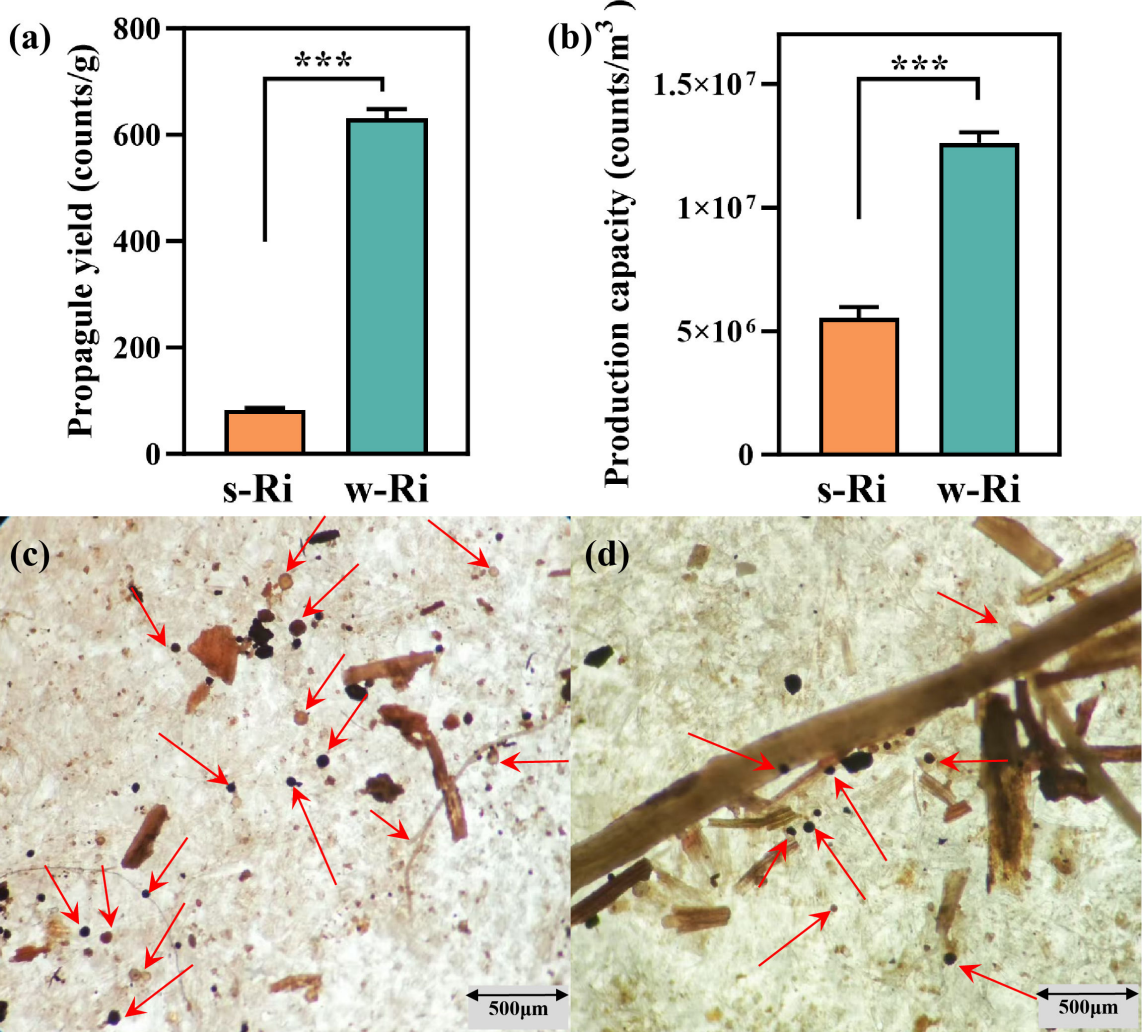
Yield of AMF propagules and the production capacity of the equipment obtained from the two production methods. (a) Fungal propagule production for both modalities; (b) the production capacity of the equipment for both modalities; (c) the spores of w-Ri inoculum; (d) the spores of s-Ri inoculum. The results are the means ± standard deviations of 5 values, and * represents differences between different Ri inoculums at the same time. *P < 0.05, **P < 0.01, ***P < 0.001.

This study sampled and quantified the propagation of the w-Ri inoculum at day 35 and the s-Ri inoculum at day 150, successfully observing distinct AMF spores and root segments (Fig. S2). The comparative analysis revealed a significantly higher number of fungal propagules in the w-Ri inoculum compared to the s-Ri inoculum (*P* < 0.001) (Fig. 2). The fungal propagule yield of the w-Ri inoculum was 7.61 times greater than that of the s-Ri inoculum. Additionally, the production capacity of the w-Ri inoculum was significantly higher than that of the s-Ri inoculum (*P* < 0.001), with a production capacity 2.28 times greater than that of the s-Ri inoculum.

### Fungal spore viability, purity, and fungal storage stability of the two Ri inoculums

**Fig 3:**
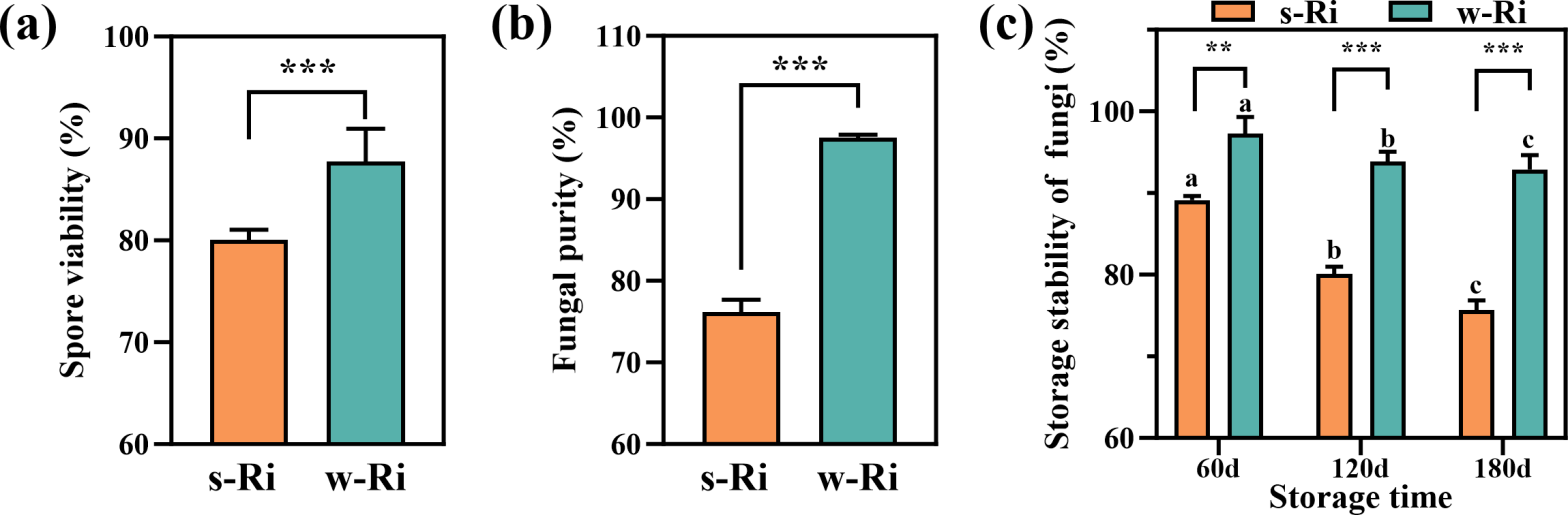
Viability of AMF fungal spores, fungal purity, and fungal storage stability obtained from the two production methods. (a) Spore viability of the two Ri inoculums; (b) fungal purity of the two Ri inoculums; (c) storage stability of the two Ri inoculums. The results are the means ± standard deviations of 5 values; there were no significant differences observed among the treatment groups containing the same letters, and * represents differences between different Ri inoculums at the same time. *P < 0.05, **P < 0.01, ***P < 0.001.

This study sampled the w-Ri inoculum at 35 days of propagation and the s-Ri inoculum at 150 days of propagation. Comparative analysis revealed that the spore viability of the w-Ri inoculum was significantly higher than that of the s-Ri inoculum (*P* < 0.001), with a 7.75% increase in spore viability for the w-Ri inoculum (Fig. 3a). Furthermore, the fungal purity of the w-Ri inoculum was also significantly greater than that of the s-Ri inoculum (*P* < 0.001) (Fig. 3b). The spore viability of the s-Ri inoculum, in comparison to the w-Ri inoculum, significantly decreased with increasing storage time for both inoculums (*P* < 0.01). However, the storage stability of spores in the w-Ri inoculum was significantly higher than that in the s-Ri inoculum (*P* < 0.001), with an 18.23% improvement in storage stability for the w-Ri inoculum at 180 days of storage.

### Mycorrhizal colonization rate and colonization of rice seedlings after inoculation with different Ri inoculums

**Fig 4:**
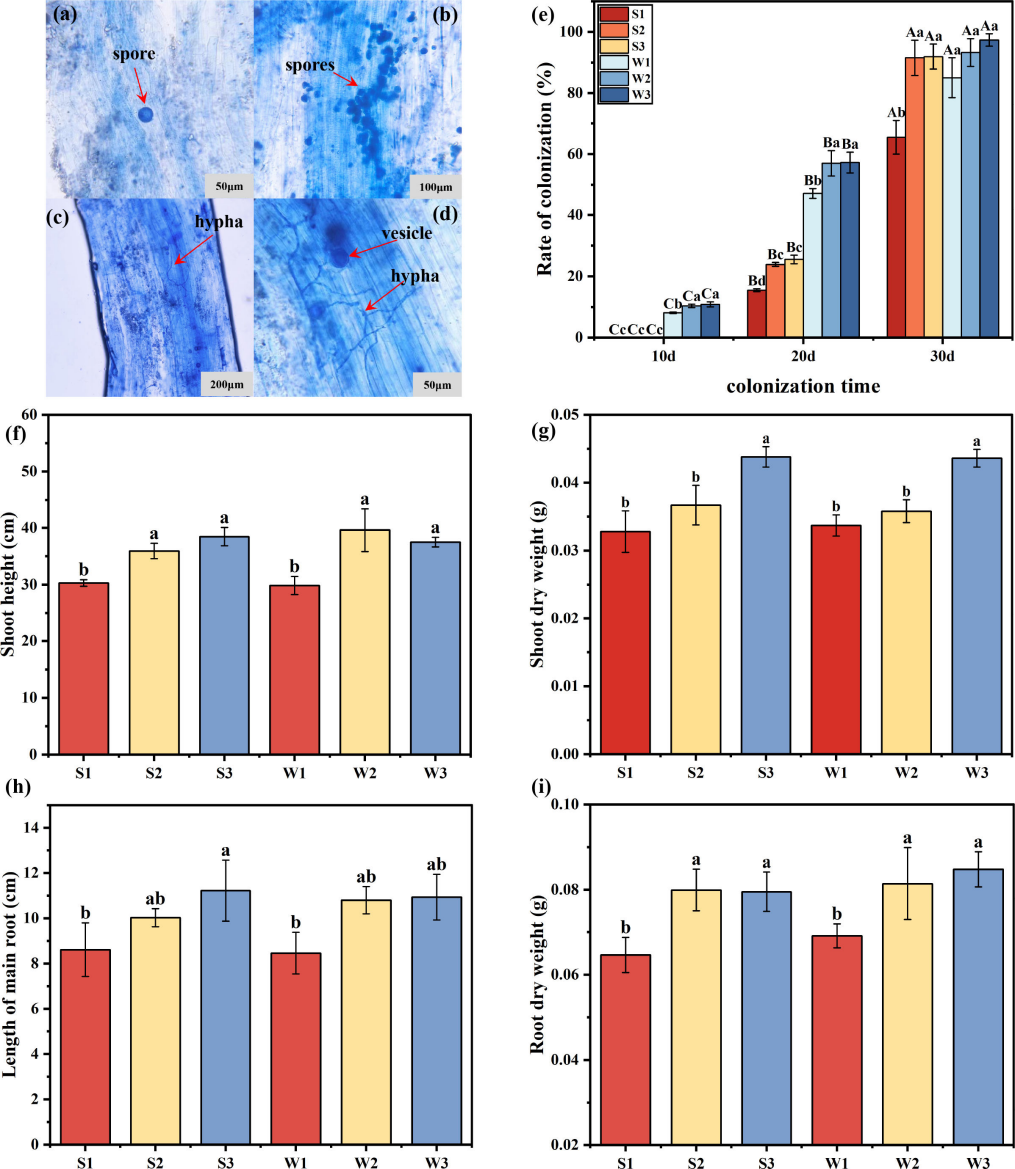
Biomass and mycorrhizal colonization rates and colonization rates of rice seedlings under a microscope. (a) Colonization in group S (400×); (b) colonization in group W (200×); (c) mycelial structure in group S (100×); (d) mycelial structure in group W (400×); (e) colonization rate of mycorrhizae in rice seedlings at different colonization times; (f) rice plant height; (g) aboveground dry weight of rice; (h) root length of rice; and (i) root dry weight of rice. The results are the means ± standard deviations of 5 values; there were no significant differences observed among the treatment groups containing the same letters, and * represents differences between different Ri inoculums at the same time. *P < 0.05, **P < 0.01, ***P < 0.001.

This experiment assessed mycorrhizal colonization in rice plants across various treatment groups. Clear Ri spores, vesicles, and intraradical hyphal structures were observed within the rice root systems of both the s-Ri and w-Ri inoculum groups (Fig. 4a through d). Moreover, the colonization rate of mycorrhiza in rice seedlings exhibited an upward trend as the culture period increased. Compared to the s-Ri inoculum, the w-Ri inoculum established symbiosis with rice seedling roots earlier (Fig. 4e). The addition of inoculum significantly influenced the biomass of the rice seedlings (*P* < 0.01).

The biomass of rice across different treatment groups was evaluated, as illustrated in Fig. 4f through i. The amount of inoculum added had a significant effect on the biomass of rice seedlings (*P* < 0.01). The shoot height and root dry weight of rice in groups S2, S3, W2, and W3 were greater than those in groups S1 and W1. Additionally, the root length and stem dry weight of rice in groups S3 and W3 were higher than those in groups S1 and W1.

### Rice root morphology after inoculation with different Ri inoculums

**Fig 5:**
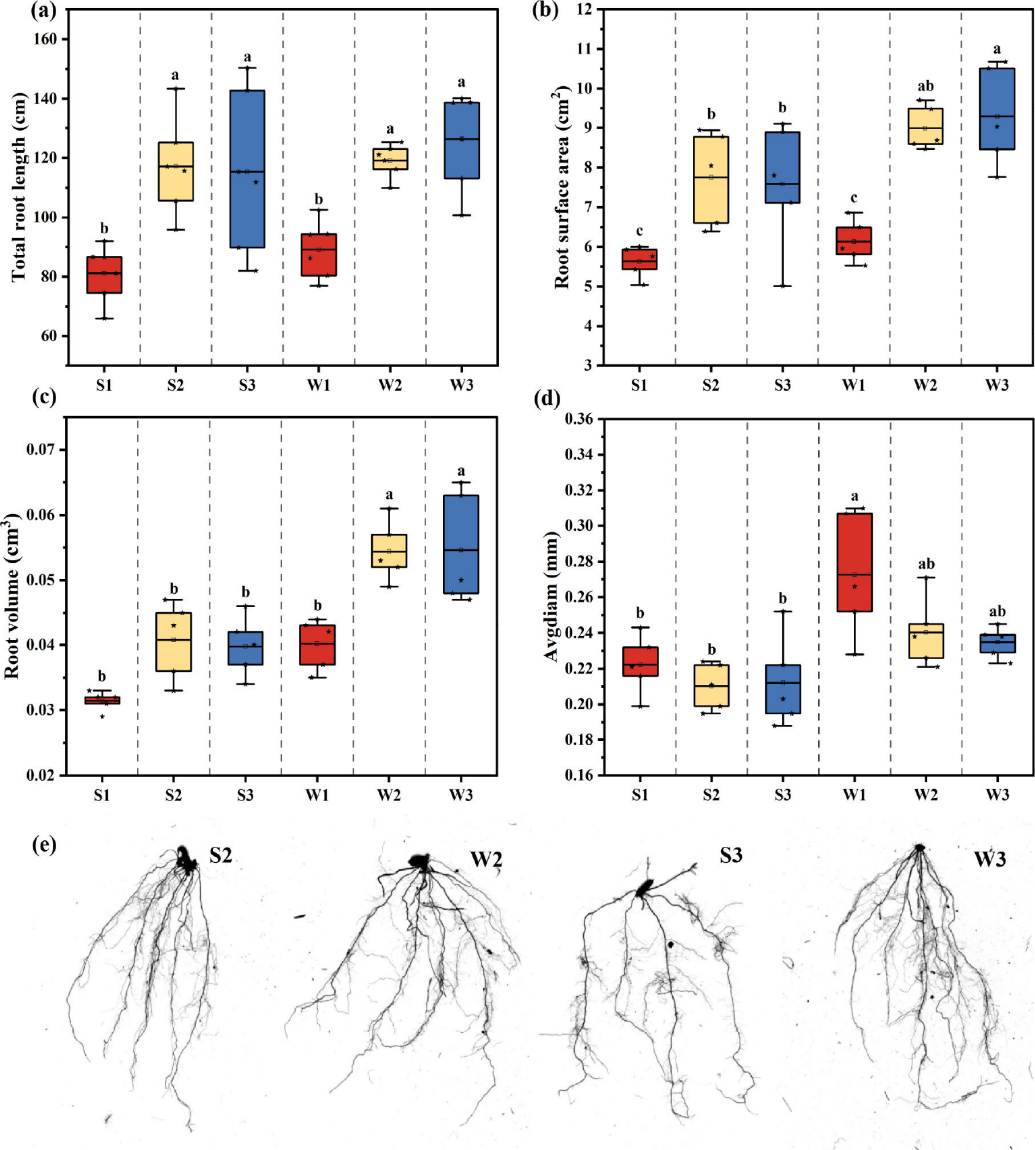
Root morphology of rice after inoculation with different Ri inoculums. (a) Total root length; (b) root surface area; (c) root volume; (d) average root diameter; (e) photographs of rice root morphology. The results are the means ± standard deviations of 5 values; there were no significant differences observed among the treatment groups containing the same letters.

Figure 5 meticulously illustrates the morphological changes in rice root systems following inoculation with various Ri inoculums. The research findings indicate that increasing the application rate of the two Ri inoculums enhanced the total root length, root surface area, and root volume of the rice seedlings. Specifically, the total root length in groups S2, S3, W2, and W3 exhibited a marked increase compared to groups S1 and W1. Additionally, the root surface area in group W3 was the most prominent among all treatment groups, surpassing that of the other groups. In terms of root volume, groups W2 and W3 also outperformed the other treatment groups.

A detailed quantitative analysis was performed on the average root diameter, number of root tips, and number of forks of rice plants after inoculation with Ri inoculums (Fig. 5d) (Fig. S3). The root diameter in group W1 was significantly greater than that in the other treatment groups (*P* < 0.01). As the application rate of Ri inoculums increased, the number of rice root tips showed a gradual upward trend. In comparing the number of root forks, groups S2, W2, and W3 outperformed group W1

### Photosynthetic gas exchange parameters, light response curves, and chlorophyll fluorescence parameters of rice after inoculation with different Ri inoculums

**Fig 6:**
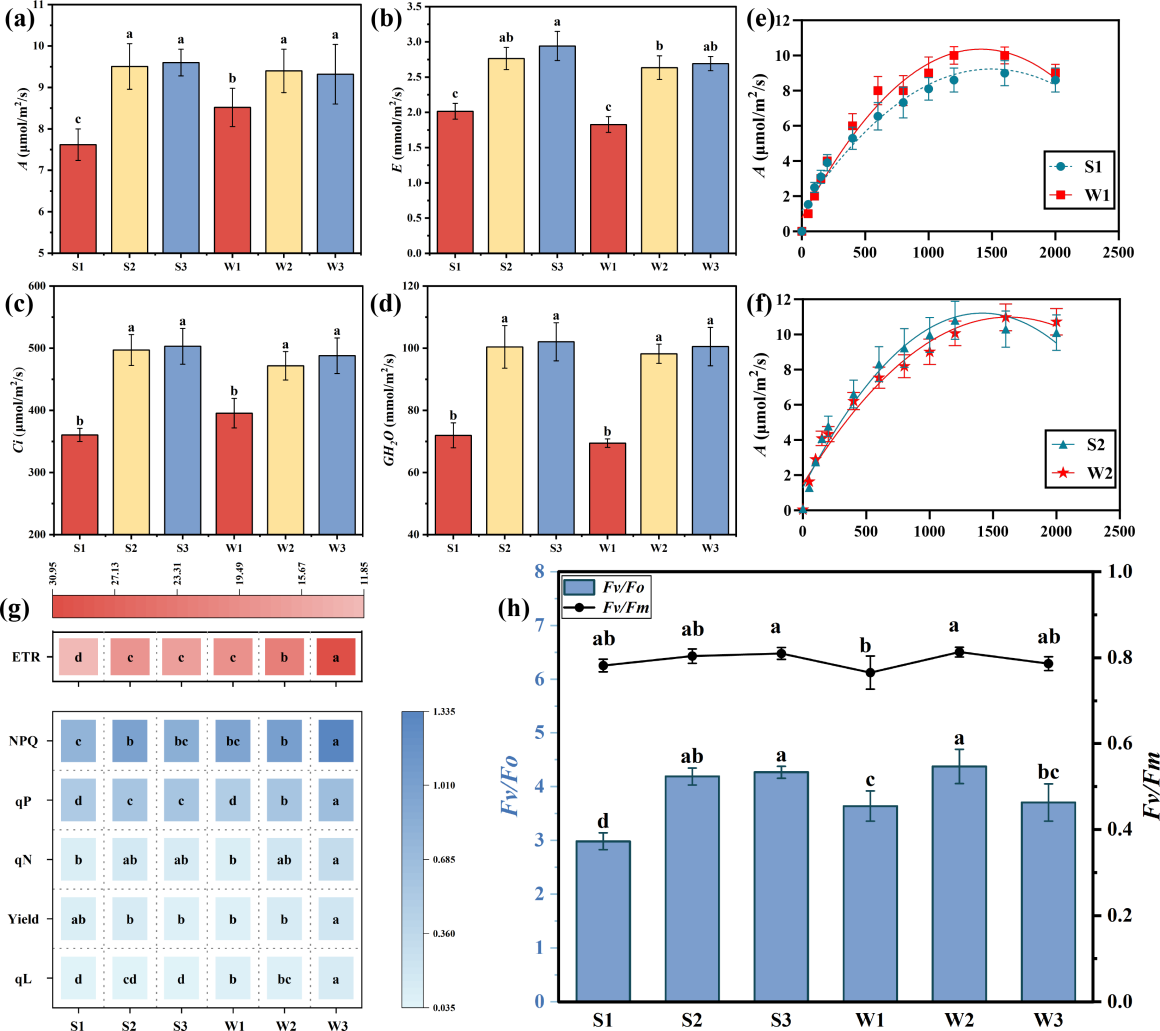
Photosynthetic gas exchange parameters, light response curves, and chlorophyll fluorescence parameters of rice in different treatment groups. (a) Net photosynthetic rate; (b) transpiration rate; (c) intercellular carbon dioxide concentration; (d) stomatal conductance; (e) light response curves of the S1 and W1 groups; (f) light response curves of the S2 and W2 groups. (g) Chlorophyll fluorescence parameters of the different treatments. (h) Changes in the photosynthetic parameters of the different treatments. *A*: net photosynthetic rate; *E*: transpiration rate*; Ci*: intercellular CO_2_ concentration; *GH*_2_*O:* stomatal conductance; *Fv/Fm*: maximum fluorescence efficiency; *Fv/Fo*: potential photochemical efficiency; *Yield: PSII* efficiency; *ETR*: apparent photosynthetic electron transfer rate; *qL*: relative component of quantum yield; *qP*: photochemical burst coefficient; *qN*: nonphotochemical burst coefficient. The results are the means ± standard deviations of 5 values; there were no significant differences observed among the treatment groups containing the same letters.

The analysis of photosynthetic gas exchange parameters in rice (Fig. 6) revealed that the net photosynthetic rate (*A*), transpiration rate (*E*), intercellular carbon dioxide concentration (*Ci*), and stomatal conductance (*GH*_2_*O*) exhibited consistent trends. Specifically, the photosynthetic gas exchange parameters of the S2, S3, W2, and W3 groups were higher than those of the S1 and W1 groups. The fitting of the trend of *A* across 12 different *PAR* gradients indicated an exponential relationship between *A* and increasing light-responsive radiation, demonstrating a strong fit (*R*^2^ > 0.95). When *PAR* ranged from 0 to 800 µmol/m^2^/s, *A* increased rapidly with rising *PAR* and plateaued once *PAR* exceeded 1,000 µmol/m^2^/s, reaching its maximum value at 1,600 µmol/m^2^/s.

We found that *NPQ, ETR, qL,* and *qP* were significantly greater in the W3 treatment (*P* < 0.01). Compared with those in the S3 treatment*, Yield, NPQ,* and *ETR* in the W3 treatment increased by 58.8%, 53.3%, and 87.6%, respectively. With the addition of Ri inoculum, the *Fv/Fo* ratio tended to increase (Fig. 6h). The *Fv/Fo* ratio in the W2 treatment was greater than that in the S1, W1, and W3 treatments (Fig. 6h), and the *Fv/Fm* ratios in the six treatments were all approximately 0.8.

### Correlations between rice physiology and Ri inoculum addition

**Fig 7:**
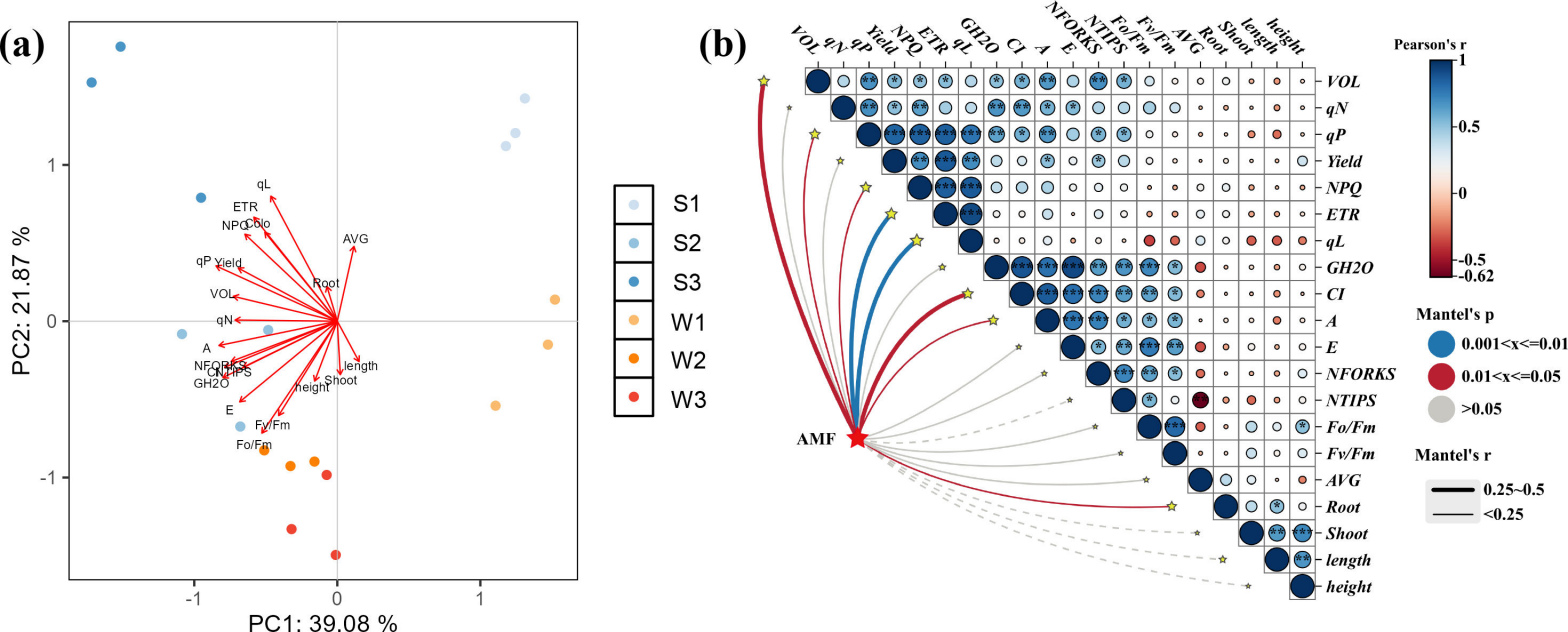
PCA and correlation analysis of various physiological growth parameters in rice. (a) PCA; (b) Mantel test. Rectangles are correlation heatmaps of various physiological parameters of rice seedlings, and the connecting lines of different colors indicate the differences in the significance levels of correlations between experimental conditions and physiological parameters. The blue line indicates a highly significant correlation (*P* < 0.01), the red line indicates a significant correlation (*P* < 0.05), and the gray line indicates no significant correlation (*P* > 0.05). *P < 0.05, **P < 0.01, ***P < 0.001.

The data on rice physiology and other metrics were analyzed using principal component analysis (PCA), with the *X* and *Y* axes representing the first (PC1) and second (PC2) principal components, respectively (Fig. 7a). The total PCA explained approximately 60.95% of the variance. The results of this study indicated that differences in the additions of Ri inoculum were primarily captured by PC1, while PC2 was more effective in distinguishing the inoculation status of the samples, highlighting clear distinctions among them. Mantel test analysis of the correlation matrix (Fig. 7b) revealed that Ri inoculum significantly influenced the chlorophyll fluorescence parameters and root morphology of the rice seedlings (*P* < 0.05). Additionally, rice photosynthetic gas exchange parameters and chlorophyll fluorescence metrics were positively correlated with root growth and development.

## DISCUSSION

### Influence of propagation methods on the mycorrhizal colonization rate and spore count of Ri inoculum

*Rhizophagus intraradices* are distinguished by their unique ability to inhabit solely within the root cells of higher plants during their entire lifecycle, a trait that allows them to prosper in hydroponic systems (23). Moreover, the root sections colonized by this fungus, in addition to its hyphae, can serve as reproductive structures. Consequently, *Rhizophagus intraradices* has been chosen as a representative organism for hydroponic systems with forthcoming plans to carry out cultivation experiments involving diverse AMF species. The colonization rate of mycorrhizae and spore density are essential determinants for evaluating the growth and development of AMF. Such measurements are frequently employed to assess the symbiotic relationship and the physiological and ecological functions between the Ri inoculum and the host plant (24). In our research, the w-Ri inoculum was found to establish mycorrhizal structures earlier with the host plant’s roots compared to the s-Ri inoculum (Fig. 1a). In comparison to the soil structure, the increased fluidity of water might enable AMF spores to contact host plant roots earlier, and the plant root system can supply oxygen and nutrients required for the germination of AMF spores (25). This contributes to their germination and expansion (26).

Moreover, the w-Ri inoculum exhibited a significantly higher spore density compared to the s-Ri inoculum (Fig. 1b). This difference may be attributed to the fact that the w-Ri inoculum is derived from hydroponics and consists entirely of AMF-colonized root segments, hyphae, and spores. In contrast, the s-Ri inoculum, which utilizes soil as a carrier, predominantly comprises soil structure, resulting in a relatively lower density of AMF spores. Additionally, effective water flow and a larger root system area of the host plants enhance spore production and dispersal (27). Furthermore, soil may harbor pathogens and other microbial competitors that adversely affect AMF growth (28). In contrast, the hydroponic environment is relatively clean, thereby mitigating these potential negative impacts. Consequently, compared to soil culture, the hydroponic method is preferable for culturing Ri inoculum, and this cultivation technique holds significant promise for future production.

### Influence of propagation methods on propagule yield and the production capacity of the equipment

The yield of fungal propagules and the production capacity of the equipment are critical parameters for evaluating the propagation status of *Rhizophagus irregularis* (Ri) inoculum, which is commonly used to assess the efficacy of new fungal cultivation methods (18). The primary objective of preparing Ri inoculum is to provide a substantial quantity of fungal propagules for plant inoculation, as the number of propagules directly influences the inoculum’s effectiveness (21). The production capacity of the equipment is evaluated to determine the efficiency of the Ri inoculum propagation system, a key factor in meeting market demands (29). Adequate inoculum ensures effective symbiosis between AMF and plant roots following inoculation, thereby promoting plant growth (30). This study revealed that the w-Ri inoculum exhibited higher fungal propagule production and superior equipment production capacity compared to the s-Ri inoculum. Consequently, the hydroponic propagation method for Ri inoculum developed in this study yielded improved results, which can advance inoculum production technology and enhance the effectiveness of mycorrhizal fungi applications in agricultural production.

### Effects of the propagation method on fungal spore viability, purity, and fungal storage stability of Ri inoculums

Fungal spore viability, purity, and storage stability are critical parameters for evaluating the quality of AMF inoculums, essential for ensuring their effectiveness and suitability (31). Spore viability directly influences the fungus’s ability to colonize plant root systems and form mycorrhizae post-inoculation, which subsequently enhances plant growth and resilience (32). It also serves as an indicator of inoculum storage stability (33). High inoculum purity minimizes the presence of non-target microorganisms, ensuring the safety of plant inoculation (34). In this study, the w-Ri inoculum demonstrated superior fungal spore viability, purity, and storage stability compared to the s-Ri inoculum. These findings indicate that the w-Ri inoculum is more adaptable and effective. This advantage may stem from hydroponic systems, which reduce AMF propagules’ exposure to environmental factors and mitigate damage from other microorganisms and harmful substances. Storage-stable inoculums maintain their efficacy across various environmental conditions, broadening their application potential. Therefore, the w-Ri inoculum designed in this study holds significant research and application prospects.

### Effects of different Ri inoculums on the rice colonization rate and biomass

The growth of rice at 35 days is rapid, allowing the plant to resist stress throughout its entire growth period. Thus, this study selected rice at this stage for measurement (35). Our findings revealed that, compared to the s-Ri inoculum, the w-Ri inoculum established symbiosis with rice seedling roots earlier (Fig. 4e). Additionally, the study found that both Ri inoculums influenced the mycorrhizal colonization rate during the early stages of rice growth. This may be attributed to the higher quantity of inoculums in the w-Ri inoculum, which facilitated easier contact with the rice roots (36). Notably, the w-Ri inoculum significantly promoted rice seedling biomass, performing as well as or even better than the normal addition of the s-Ri inoculum when diluted. These results provide a solid theoretical foundation for the development of Ri inoculum production methods and its application in rice cultivation, presenting a potential strategy for enhancing rice biomass in the future.

### Effects of different Ri inoculums on rice root morphology

Adaptive changes in root morphology are crucial for plant survival and reproduction (37). The morphology of the root system determines a plant’s ability and efficiency in absorbing water and mineral nutrients (36, 38). Moreover, the root system is a significant site for synthesizing certain plant hormones that regulate plant growth and development (39). In this study, significant changes in rice root morphology were observed with increasing addition, and the effects of w-Ri inoculum were similar to those of s-Ri inoculum. This similarity may be attributed to AMF promoting plant root growth and branching by interacting with the root system and influencing the hormonal balance within the plant (40, 41). These findings suggest that the w-Ri inoculum holds significant advantages for future agricultural production activities.

### Effects of different Ri inoculums on photosynthetic gas exchange parameters and chlorophyll fluorescence parameters in rice

Photosynthesis serves as the fundamental basis for plant survival and development (42, 43). Additionally, photosynthetic gas exchange parameters are crucial metrics for evaluating plant growth capabilities (44). The current study discovered that the impact of the w-Ri inoculum was comparable to, or even exceeded, that of the s-Ri inoculum. This might be due to the purity of the w-Ri inoculum, which mitigates the impact of other microorganisms on rice growth (45, 46), thereby maintaining a potent inoculation effect.

Chlorophyll fluorescence parameters in plants serve as significant metrics for assessing photosynthetic status and physiology (47, 48). The *Fv/Fo* and *Fv/Fm* parameters, respectively, represent the potential activity and maximum photochemical quantum efficiency of dark-adapted *PSII* reaction centers (49, 50). Meanwhile, the combined interpretation of *Yield, ETR, qP, qN,* and *qL* provides insights into plant utilization and regulation of light energy during photosynthesis (51). These parameters are pivotal in assessing the photosynthetic function and physiological status of plants (52). The findings suggest that the w-Ri inoculum enhances the photosynthetic regulation and potential stress tolerance of rice seedlings, thereby facilitating improved rice growth. Future research should concentrate on the development of innovative Ri inoculums, comprehensive analysis of their molecular mechanisms in relation to plant photosynthesis, and the potential application of these interactions to augment crop yield and ecosystem stability.

### Conclusion

A significantly higher quality of Ri inoculum was obtained through hydroponics. Compared to the s-Ri inoculum, the w-Ri inoculum demonstrated greater propagule yield, equipment productivity, spore viability, fungal purity, and storage stability. The w-Ri inoculum formed mycorrhizal structures earlier in the rice root system, as evidenced by the effects of rice inoculation. Additionally, the w-Ri inoculum promoted rice growth more effectively than the s-Ri inoculum. Consequently, the w-Ri inoculum shows superior application effects and broader application scenarios. This study provides a theoretical basis and data support for establishing a hydroponic Ri inoculum propagation system and introduces a new model for the application of Ri inoculum in agricultural production.

## MATERIALS AND METHODS

### Experimental materials

The arbuscular mycorrhizal fungus selected for this study was *Rhizophagus intraradices* (Ri, preservation number CGMCC No. 10607). Using sorghum as the host, arbuscular mycorrhizal fungi were propagated in pots, resulting in an approximate spore density of 35 spores per gram. The spores of the arbuscular mycorrhizal fungi were then collected through a density gradient sucrose centrifugation method.

The host plant selected is sorghum, which is characterized by rapid growth and a well-developed root system. For the inoculation study, the rice (*Oryza sativa* L.) variety selected was Longdao 18, which was acquired from the Heilongjiang Academy of Agricultural Sciences.

### Experimental design

#### Propagation of s-Ri inoculum

A mixture of soil, sand, and vermiculite at a ratio of 5:3:2 was divided into individual culture pots to create the Ri culture substrate. The soil’s physicochemical properties included a pH of 5.9, organic matter content of 36.52 mg/kg, total nitrogen concentration of 1.79 g/kg, total phosphorus concentration of 0.75 g/kg, and potassium concentration of 2.49%. Each pot was thoroughly watered and left to stand for 1 day. Sorghum seeds were soaked in a 0.3% potassium permanganate solution by weight for 30 min and then rinsed with water. Subsequently, the seeds were soaked in water at 20–30℃ for 24 h to promote germination. 500 Ri spores were mixed thoroughly with the cultivation substrate. Germinated seeds were then sown on the substrate surface and covered with a 1 cm layer of soil. After 150 days of growth, the sorghum’s stems and leaves were removed, and the infection rate was checked. The sorghum roots were mixed with the growth medium, air-dried (40℃, 72 h), and ground to obtain the s-Ri inoculum. The main part of the s-Ri inoculum was soil, and the rest was colonized sorghum root segments and Ri spores.

#### Propagating w-Ri inoculum

**Fig 8:**
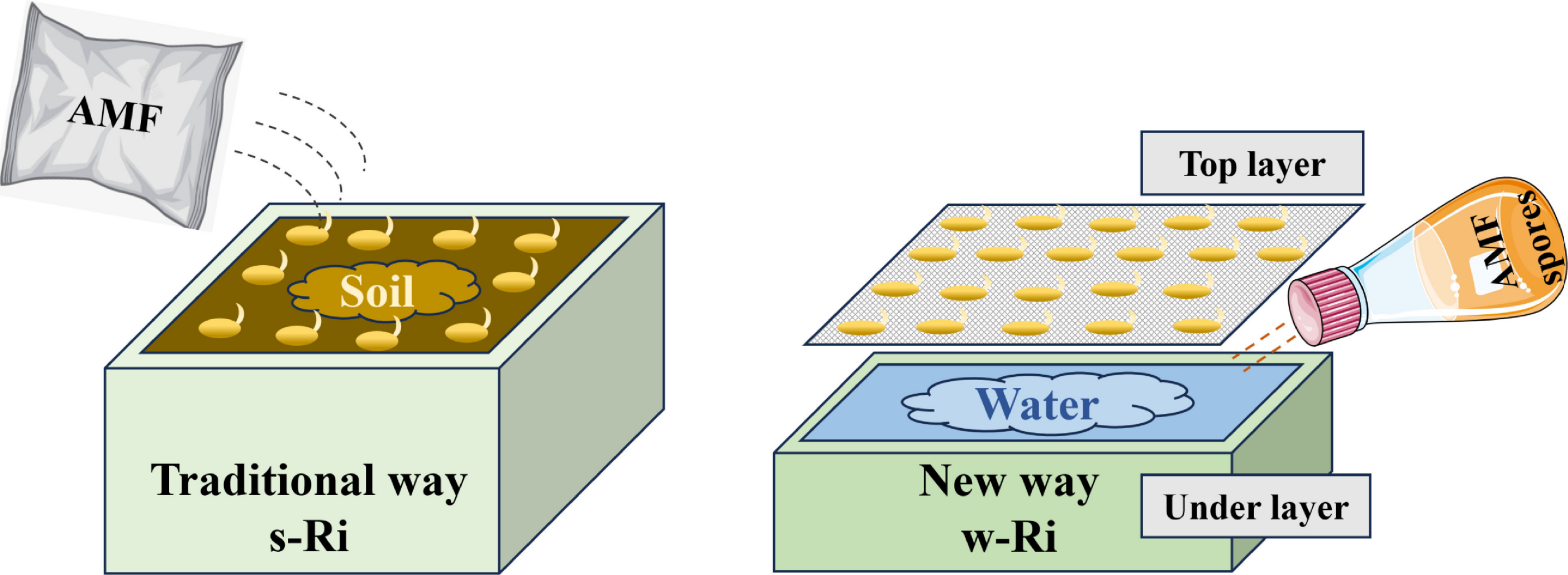
Soil propagation method and hydroponic propagation method for Ri inoculum assessment of inoculation effect.

In each cultivation container, 500 spores of *Rhizophagus intraradices*, isolated and enriched from root inocula, are added to the lower layer and thoroughly mixed with water. The cultivation container consists of a rectangular dual-layer structure made of polypropylene. The upper layer features a porous partition that allows plant roots to penetrate, while the lower layer contains a growth medium such as distilled water (Fig. S4). The dimensions of the container are 25 cm in length, 20 cm in width, and 15 cm in height. Water is then added to slightly submerge the upper cultivation plate (Fig. 8). The water is filled to slightly above the level of the upper culture plate. The seed treatment method was identical to that used for the s-Ri inoculum. Post-germination, the sorghum seeds are evenly spread across the upper tray section. After 35 days of growth, the sorghum’s stem and leaf components are removed, and the colonization rate is assessed. The w-Ri inoculum comprises a rhizosphere mix of colonized sorghum root segments, Ri spores, and mycelium.

The inoculation effects of two distinct Ri inoculants at varying addition levels were assessed using rice as the host plant. The w-Ri inoculant was diluted to a tenth of its original concentration (vol/vol) with distilled water prior to use, while a corresponding volume of distilled water was added to the s-Ri inoculant group. Thus, the experimental treatments for the s-Ri inoculant (S) were as follows: 1% (S1), 5% (S2), and 10% (S3) by volume. For the w-Ri inoculant (W), the treatments were as follows: 1% (W1), 5% (W2), and 10% (W3) by volume. The goal was to compare the inoculation effects between the s-Ri and w-Ri inoculants.

The Ri inoculant for each treatment was incorporated into the rice seedling substrate according to its mass percentage and mixed thoroughly. Each inoculum treatment is replicated in five pots, with six seedlings per pot. The pots are arranged in a randomized complete block design to mitigate edge effects that may influence the experimental outcomes. Each pot contains 0.75 kg of substrate soil. The rice was housed in an artificial climate chamber (R-1000E4DW-CO2-N2O, China) and cultured under conditions of a 14 h light and 10 h dark cycle, maintaining a humidity of 70%. The soil’s physicochemical characteristics included a pH of 5.9, an organic matter content of 36.52 mg/kg, a total nitrogen concentration of 1.79 g/kg, a total phosphorus concentration of 0.75 g/kg, and a potassium concentration of 2.49 g/kg.

### Evaluation of Ri-tested inoculum efficiency

#### Total mycorrhizal rate determination and AMF spore quantification

##### Ri inoculum colonization rate

On days 14, 21, 28, and 35 after propagating the Ri inoculum, 50 root segments of sorghum are randomly selected at each time point. These segments are then cut into small pieces ranging from 0.5 cm to 1 cm in length. The colonization rate is calculated as follows (53):

Root colonization (%) = (number of arbuscular mycorrhiza-positive segments)/total number of segments studied × 100%.

##### Ri inoculum spore density

In order to compare the final quality of the inoculum produced by the two propagation methods, the w-Ri inoculum harvested on the 35th day and the s-Ri inoculum collected on the 150th day were selected for measurement of the spore count of the Ri inoculum (54). For each treatment group, 2 g of each Ri inoculum is collected from the corresponding pots. The samples are then ground and sieved through a 200-mesh screen, with the residual material collected and transferred into centrifuge tubes for further analysis. The residue on the sieve was placed into a centrifuge tube, an equal volume of 50% sucrose solution was added, and the mixture was centrifuged at 3,000 r/min for 20 min. Collect the supernatant and then filter it through filter paper to obtain the spores. And the spores were counted under a stereomicroscope (Leica EZ4 W, Germany).

### Propagule yield and the production capacity of the equipment

Both spores and root fragments are considered propagules. The method for determining the propagule yield involves staining and observing the mycorrhizal fragments, with the treatment procedure described above (55):

Propagule yield (counts/g) = (spores + root fragments)/weight of (spores + root fragments + the carrier material).

We define the equipment production efficiency as the number of propagules produced by the experimental apparatus per unit volume within a specified time frame. To investigate the final inoculum quality of the two propagation methods, three randomly selected pots containing w-Ri inoculum on day 35 and s-Ri inoculum on day 150 were used for propagule quantification, following the same method as described above (21):

Production capacity of the equipment (counts/m^3^) = (number of propagules)/(equipment size).

### Spore viability

The calculation of spore viability was performed via the MTT [3-(4,5-dimethylthiazol-yl)-2,5-diphenyl-2H-tetrazolium bromide] staining method (56). The measurement was conducted using the w-Ri inoculum taken on the 35th day and the s-Ri inoculum taken on the 150th day. Three pots were randomly selected from each of the two Ri inoculum treatments, and 100 spores were collected from each pot. The spores were placed in Petri dishes with a diameter of 30 mm. Each sample was performed with three biological replicates. Viable spores appear pink or red under yellow light, whereas nonviable spores appear dark or show no staining under yellow light.

Spore viability (%) = (number of spores stained red or pink)/(total number of spores) × 100.

### Fungal purity

One gram of each of the two Ri inoculants was subjected to gradient dilution and then spread on PDA media plates. Fungal contamination was checked via stereomicroscopy (57).

Fungal purity (%) = (number of Ri spores)/(number of fungal colonies + number of spores) × 100

### Spore storage stability

To determine the storage stability of the two types of Ri inoculum, the remaining samples of both inoculum types were stored under identical conditions in the same cool, dry warehouse. At time intervals of 60 days, 120 days, and 180 days, the calculation method is the same as described in “Spore viability,” above(58).

Storage stability of fungi (%) = (number of surviving spores)/(number of spores surviving before storage) ×100

### Evaluation of the inoculation effectiveness of the Ri inoculum

#### Determination of the rice colonization rate, biomass, and root system status

##### Rice colonization rate

At 10 days, 20 days, and 30 days of rice cultivation, the colonization rate measurement procedure was repeated as described in formula (1), and the colonization rate was calculated.

In each treatment, five rice plants were randomly selected, and their above-ground and root sections were separated. After thorough cleaning, the shoot height and root length were measured. The rice root systems of each treatment were scanned via a root scanner (LA2400 Scanner [12000XL], China) for morphological analysis, and then the morphological data were analyzed with WinRHIZO software. Finally, the analyzed aboveground parts and roots of the rice plants were placed into an oven, the green environment was killed at 105°C for 30 min, and then the plants were dried at 70°C until a constant weight was reached to measure the dry weight of the rice.

### Measurement of rice photosynthetic gas exchange parameters, chlorophyll fluorescence parameters, and light response curves

A GFS-3000 portable photosynthesis‒fluorescence measurement system (Heinz Walz GmbH, Germany) was used to measure the photosynthetic gas exchange parameters, chlorophyll fluorescence parameters, and light response curves of the rice plants at 30 days. The measurements should be conducted between 9:00 and 11:00 AM on a clear morning (53). The light source selected for measuring photosynthetic gas exchange parameters was red–blue light, with an illumination intensity of 1,000 µmol/m²/s, and the photosynthetically active radiation (*PAR*) was 1,000 µmol/m²/s. The concentration was controlled via a CO_2_ gas cylinder at 420 µmol/s. The measured parameters included the net photosynthetic rate (*A*), stomatal conductance to water vapor (*GH*_2_*O*), intercellular CO_2_ concentration (*Ci*), and transpiration rate (*E*).

Prior to measuring the chlorophyll fluorescence parameters of the rice, the plants were subjected to a 30 min dark adaptation period, followed by measurement. The parameters measured included the maximum fluorescence (*Fm*), minimum fluorescence (*Fo*), and maximum quantum efficiency of photosystem II (*PSII*) (*Fv/Fm*). The potential photochemical efficiency (*Fv/Fo*) was then calculated. At this point, the plants have fully adapted to the light, with all primary electron acceptors in a reduced state. The measurement indices include the PSII efficiency (*Yield*), apparent photosynthetic electron transport rate (*ETR*), photochemical quenching coefficient (*qP*), nonphotochemical quenching coefficient (*qN*), and a relative component of quantum yield (*qL*) (59).

Before the light response curve was measured, the rice plants were subjected to a 30 min light induction at an intensity of 1,400 µmol/m²/s to ensure that the photosynthetic capacity of the plants was fully expressed. The photosynthetically active radiation (PAR) was set at 2,000, 1,600, 1,200, 1,000, 800, 600, 400, 200, 150, 100, 50, and 0 µmol/m²/s (12 gradients in total). The light response curves were fitted via the rectangular hyperbola correction model (60).

### Data processing and analysis

The data were processed and statistically analyzed via SPSS 25 (SPSS, Chicago, IL, USA) and plotted via the Origin2021PRO (Origin Lab-COR, Northampton, MA, USA) and ggplot2 packages (Wickham, 2016) in R software (4.3.2) (R Core Team, 2023). The data were tested for conformity to a normal distribution, and those that did not conform were log-transformed. The significance of the treatments was tested via one-way ANOVA and two-way ANOVA, and the significance of the differences between the groups was tested via the Tukey test at the 0.05 level. PCA and the Mantel test were employed to assess the impact of Ri inoculants on the growth of rice.

## Data Availability

The data will be made available upon request.

## SUPPLEMENTAL MATERIAL

Supplemental figures aem.00558-25-s0001.docx Figures S1 to S4.

Supplemental tables aem.00558-25-s0002.docx Tables S1 and S2.

ASM does not own the copyrights to Supplemental Material that may be linked to, or accessed through, an article. The authors have granted ASM a non-exclusive, world-wide license to publish the Supplemental Material files. Please contact the corresponding author directly for reuse.
