# Supplementary material for: Evaluation of the efficient propagation of Rhizophagus intraradices and its inoculation effects on rice
Source: Appl Environ Microbiol. 2025 Jun 24;91(7):e00558-25. doi: 10.1128/aem.00558-25 (PMC12285234; doi:10.1128/aem.00558-25)
Supplement: Supplemental figures — Figures S1 to S4. [file aem.00558-25-s0001.docx]

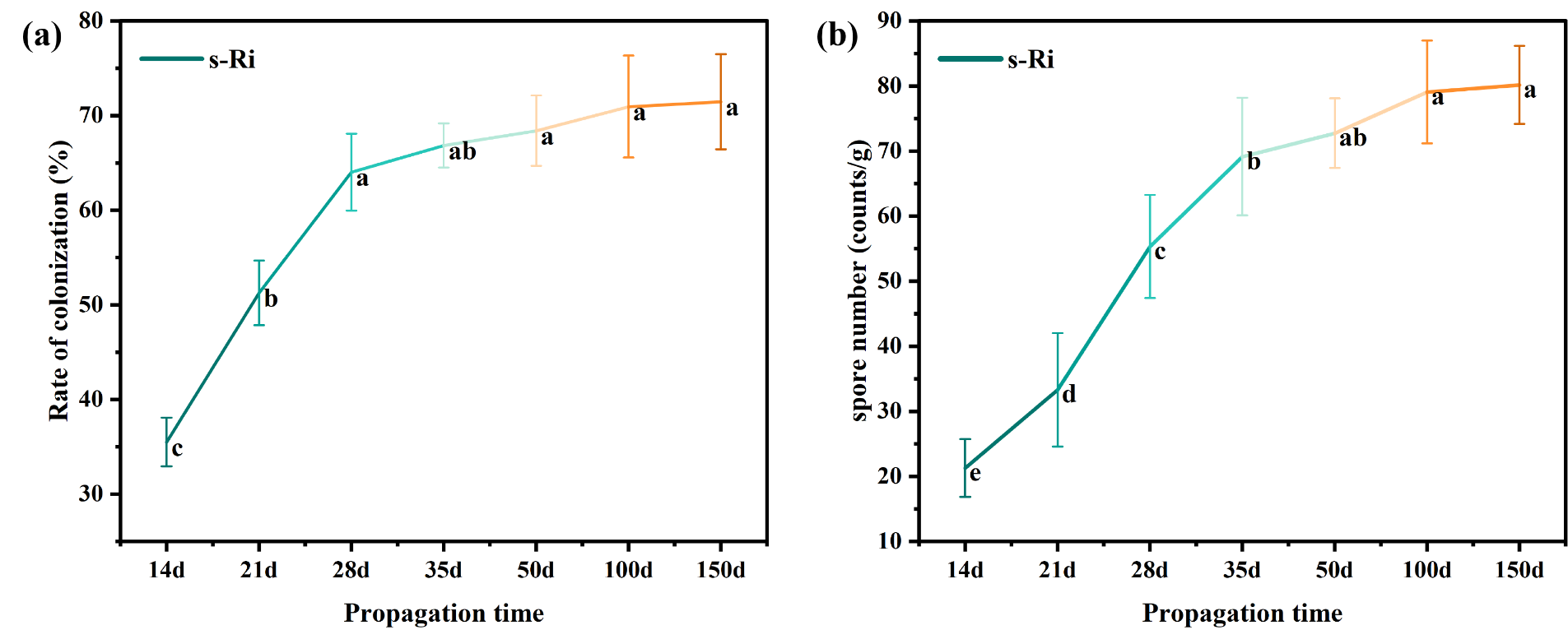


**Fig. S1 Colonization rate and spore number of s-Ri inoculum at different times**

Note: (a) Colonization rate; (b) spore number. Results are the mean ± standard deviation of 5 values, different letters represent differences between different times *P*<0.05.


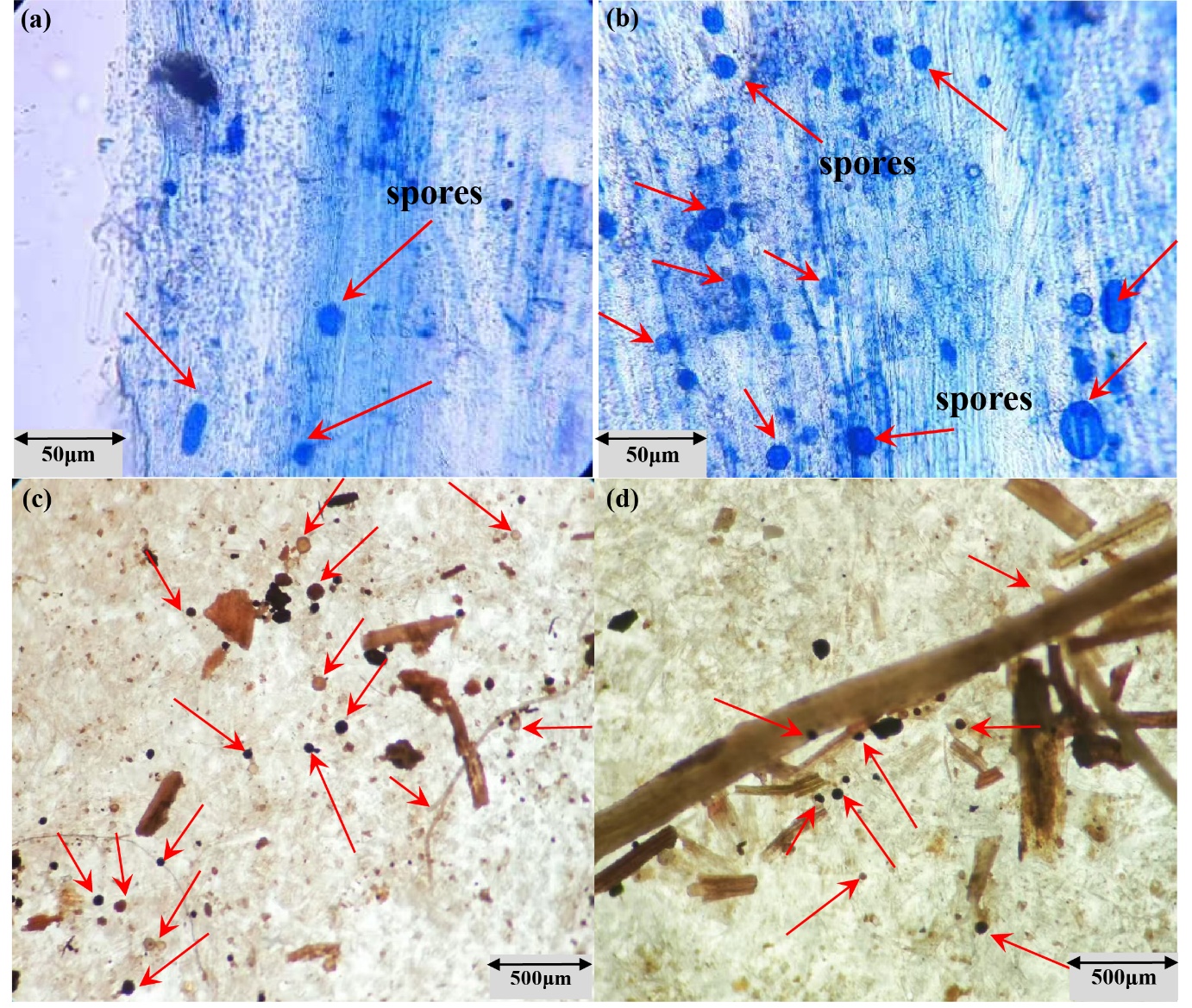


**Fig. S2 Colonization rate and spore number of s-Ri inoculum at different times**

Note: (a) Colonization of root segments by the s-Ri inoculum; (b) Root segment colonization by the w-Ri inoculum; (c) Spores and mycorrhizal fragments of w-Ri inoculum; (d) Spores and mycorrhizal fragments of s-Ri inoculum.


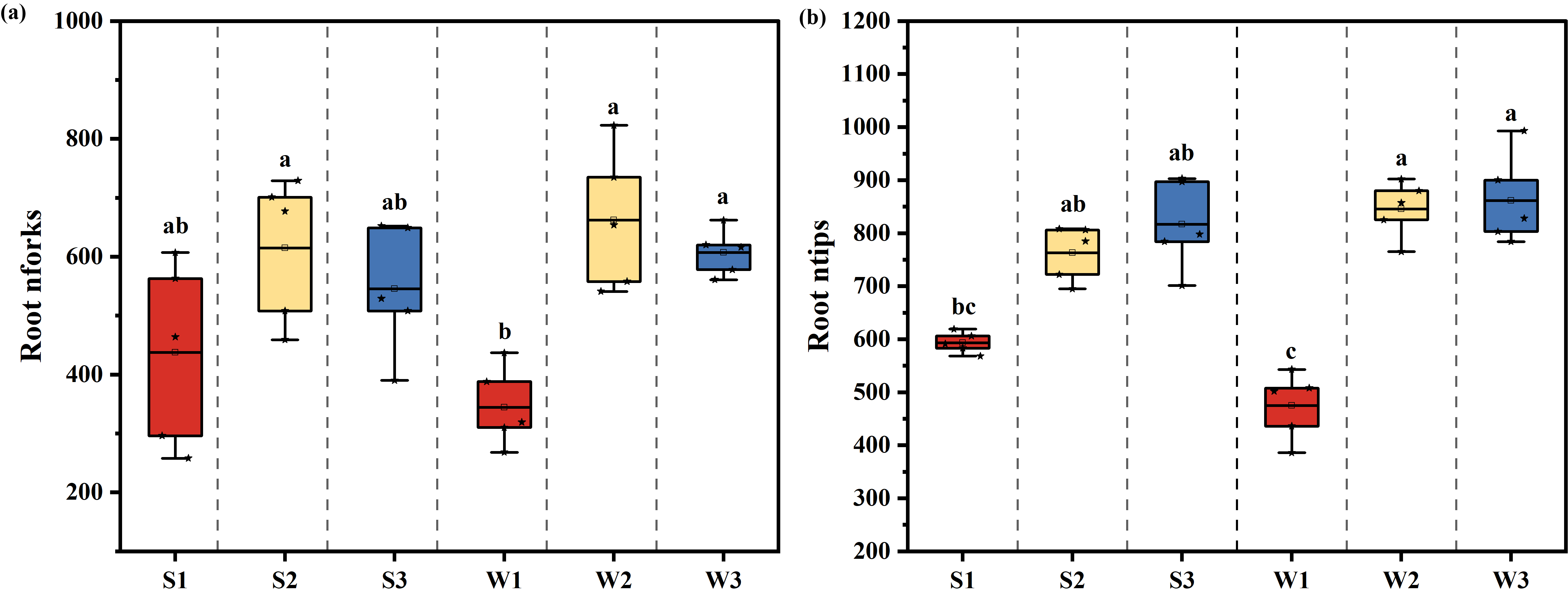


**Fig. S3 Photographs of rice root morphology**

Note: (a) root nforks; (b) root ntips. Results are the mean ± standard deviation of 5 values, different letters represent differences between different times *P*<0.05.


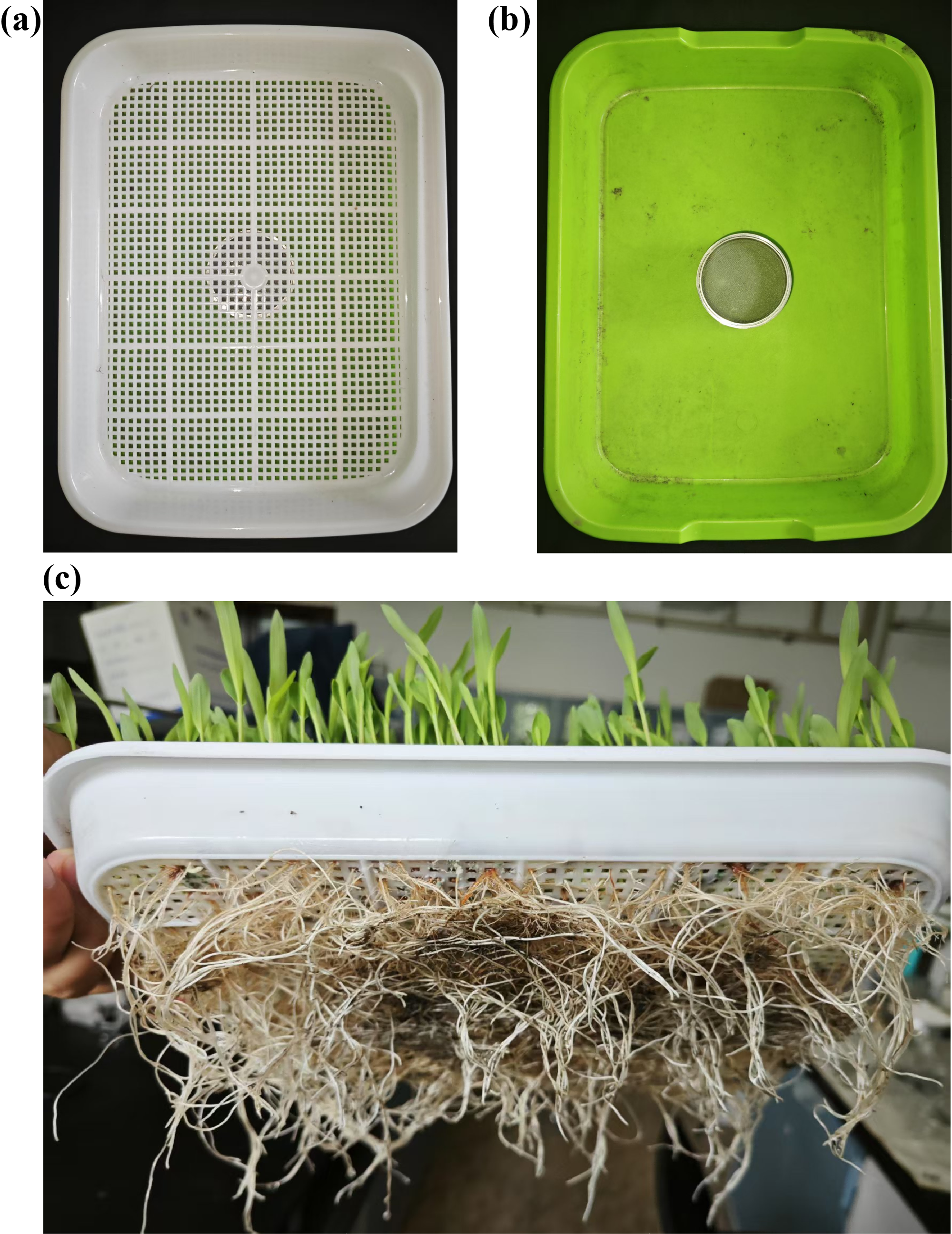


**Fig. S4 Diagram of hydroponic AMF culture device**

Note: (a) top layer of culture vessel; (b) Bottom layer of culture vessel. ; (b) Cultivation Diagram.
