# Supplementary material for: Evaluation of the efficient propagation of Rhizophagus intraradices and its inoculation effects on rice
Source: Appl Environ Microbiol. 2025 Jun 24;91(7):e00558-25. doi: 10.1128/aem.00558-25 (PMC12285234; doi:10.1128/aem.00558-25)
Supplement: Supplemental tables — Tables S1 and S2. [file aem.00558-25-s0002.docx]

**Table S1 Abbreviations**

| Serial number | abbreviations | meanings |
| --- | --- | --- |
| 1 | AMF | Arbuscular mycorrhizal fungi |
| 2 | Ri | *Rhizophagus intraradices* |
| 3 | *A* | Net photosynthetic rate |
| 4 | *E* | Transpiration rate |
| 5 | *Ci* | Intercellular CO_2_ concentration |
| 6 | *GH_2_O* | Stomatal conductance |
| 7 | w-Ri | water culture inoculum |
| 8 | s-Ri | soil-based inoculum |
| 9 | PAR | photosynthetically active radiation |
| 10 | *Fm* | maximum fluorescence |
| 11 | *Fo* | minimum fluorescence |
| 12 | *Fv/Fm* | maximum quantum efficiency of photosystem II |
| 13 | *Fv/Fo* | The potential photochemical efficiency |
| 14 | *Yield* | *PSII* efficiency |
| 15 | *ETR* | apparent photosynthetic electron transport rate |
| 16 | *qP* | photochemical quenching coefficient |
| 17 | *qN* | nonphotochemical quenching coefficient |
| 18 | *qL* | the relative component of quantum yield |
| 19 | PCA | Principal Component Analysis |

**Table S2 Colonization intensity**

| treatment group | colonization intensity |
| --- | --- |
| w-Ri | +++ |
| s-Ri | ++ |
| W1 | ++ |
| W2 | +++ |
| W3 | ++++ |
| S1 | + |
| S2 | ++ |
| S3 | +++ |

Notes: There are 5 levels of colonization intensity, with “+” being the lowest and “+++++” being the highest.
